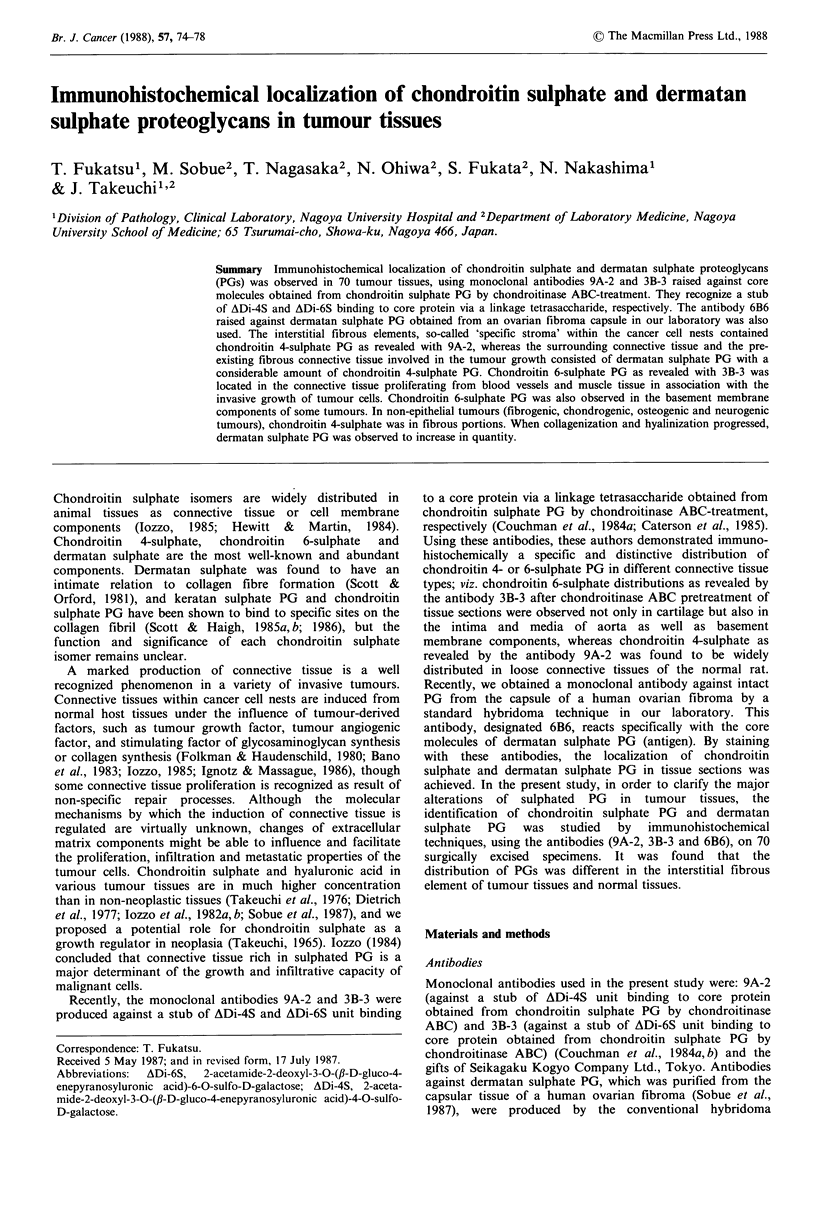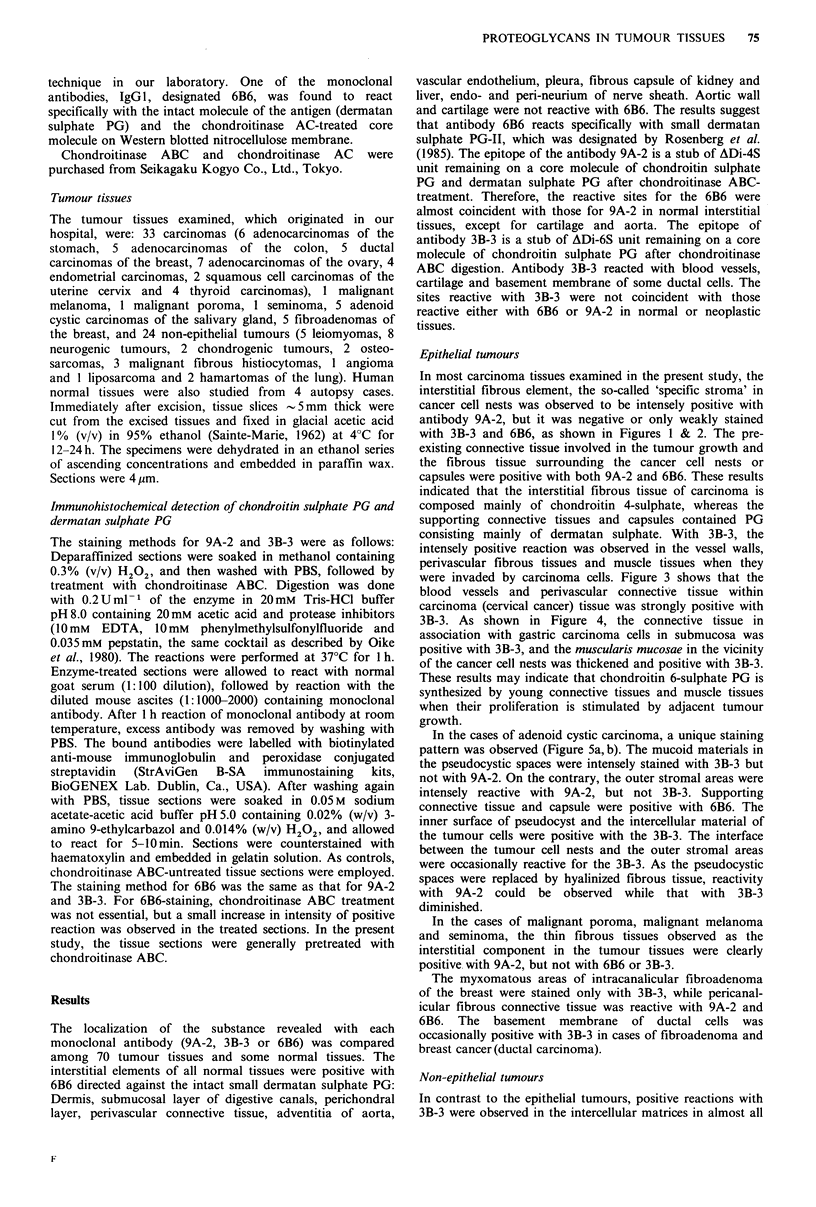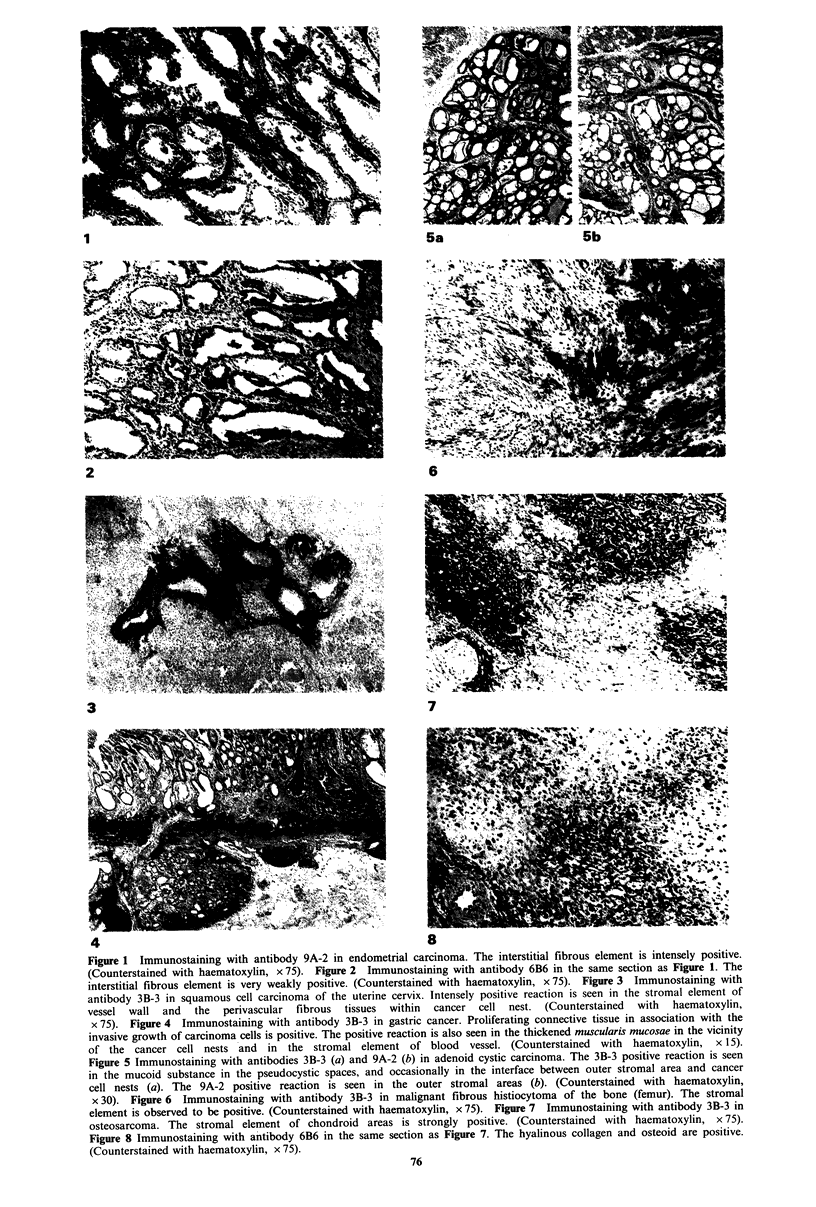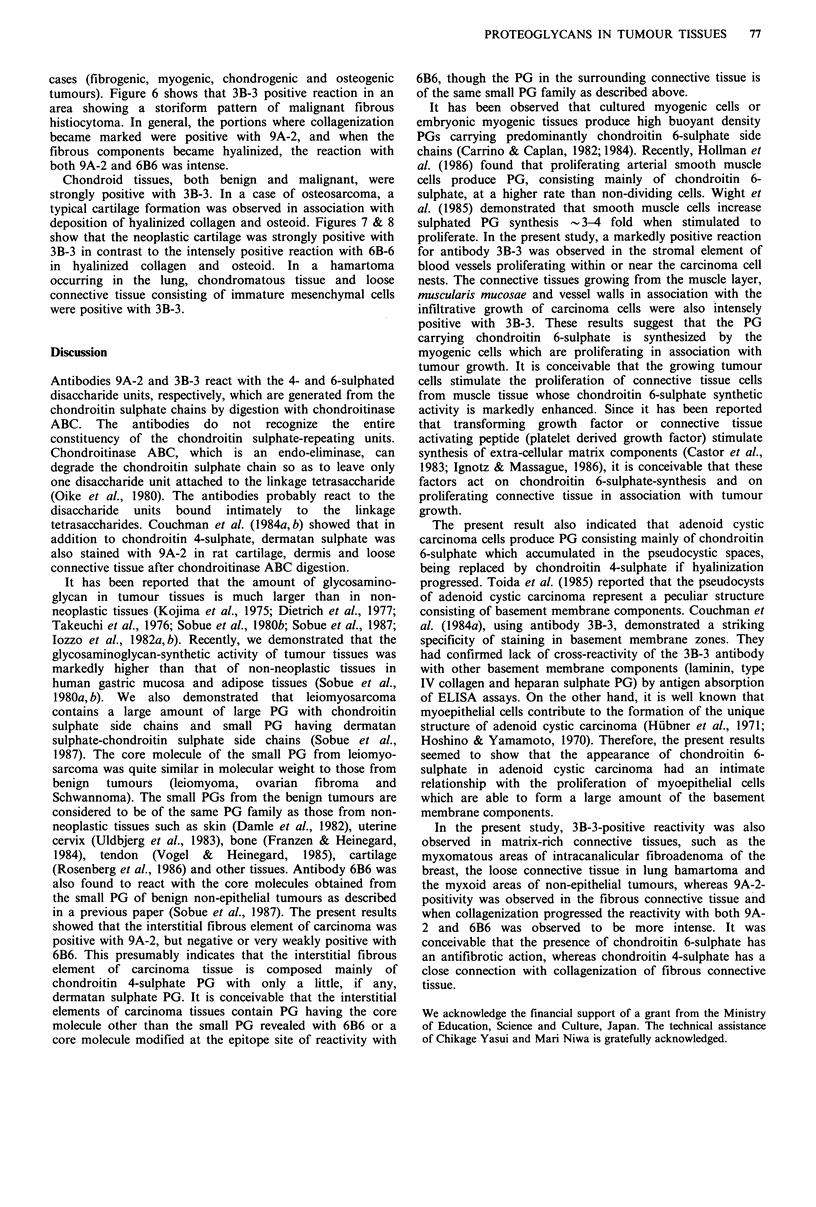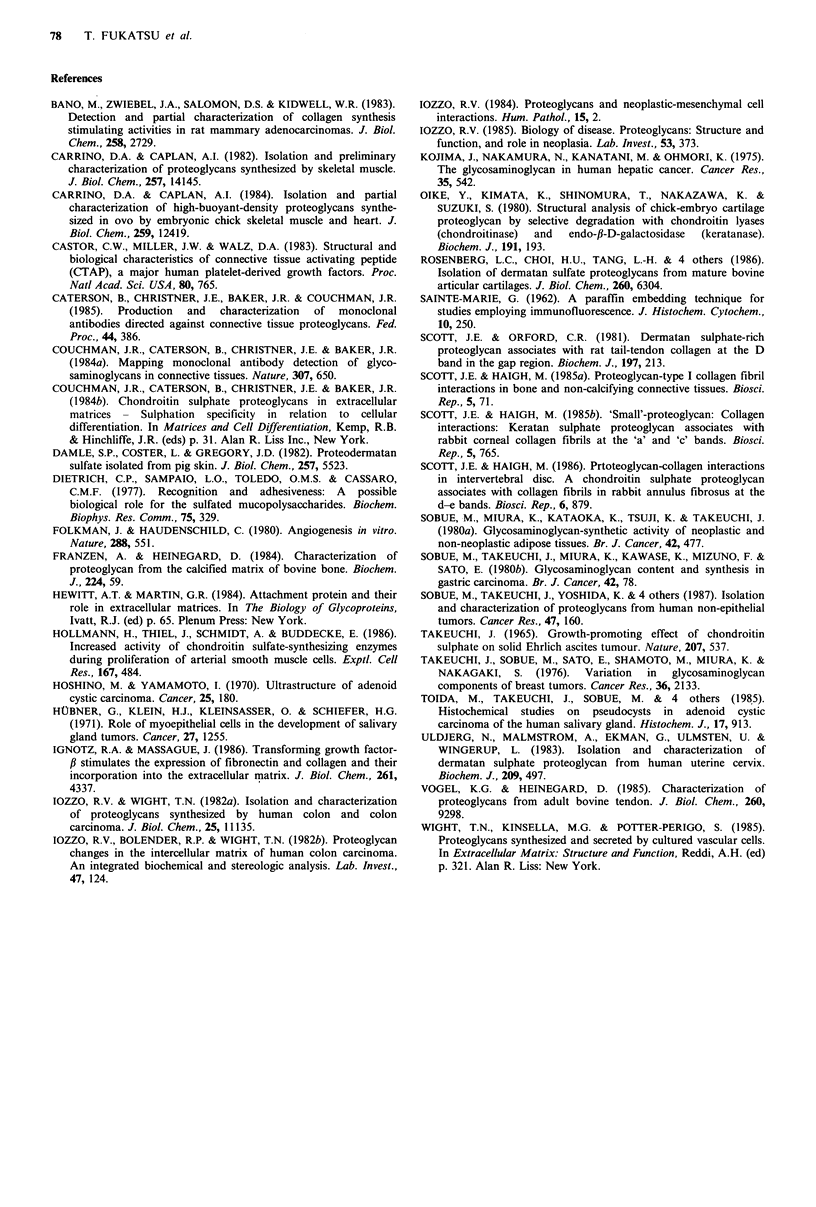# Immunohistochemical localization of chondroitin sulphate and dermatan sulphate proteoglycans in tumour tissues.

**DOI:** 10.1038/bjc.1988.12

**Published:** 1988-01

**Authors:** T. Fukatsu, M. Sobue, T. Nagasaka, N. Ohiwa, S. Fukata, N. Nakashima, J. Takeuchi

**Affiliations:** Division of Pathology, Nagoya University Hospital, Japan.

## Abstract

**Images:**


*Br. J. Cancer* (1988), **57**, 74–78 © The Macmillan Press Ltd., 1988

### Immunohistochemical localization of chondroitin sulphate and dermatan sulphate proteoglycans in tumour tissues

T. Fukatsu[1], M. Sobue[2], T. Nagasaka[2], N. Ohiwa[2], S. Fukata[2], N. Nakashima[1] & J. Takeuchi[1,2]

[1]*Division of Pathology, Clinical Laboratory, Nagoya University Hospital and* [2]*Department of Laboratory Medicine, Nagoya University School of Medicine; 65 Tsurumai-cho, Showa-ku, Nagoya 466, Japan.*

**Summary** Immunohistochemical localization of chondroitin sulphate and dermatan sulphate proteoglycans (PGs) was observed in 70 tumour tissues, using monoclonal antibodies 9A-2 and 3B-3 raised against core molecules obtained from chondroitin sulphate PG by chondroitinase ABC-treatment. They recognize a stub of ΔDi-4S and ΔDi-6S binding to core protein via a linkage tetrasaccharide, respectively. The antibody 6B6 raised against dermatan sulphate PG obtained from an ovarian fibroma capsule in our laboratory was also used. The interstitial fibrous elements, so-called 'specific stroma' within the cancer cell nests contained chondroitin 4-sulphate PG as revealed with 9A-2, whereas the surrounding connective tissue and the pre-existing fibrous connective tissue involved in the tumour growth consisted of dermatan sulphate PG with a considerable amount of chondroitin 4-sulphate PG. Chondroitin 6-sulphate PG as revealed with 3B-3 was located in the connective tissue proliferating from blood vessels and muscle tissue in association with the invasive growth of tumour cells. Chondroitin 6-sulphate PG was also observed in the basement membrane components of some tumours. In non-epithelial tumours (fibrogenic, chondrogenic, osteogenic and neurogenic tumours), chondroitin 4-sulphate was in fibrous portions. When collagenization and hyalinization progressed, dermatan sulphate PG was observed to increase in quantity.

Chondroitin sulphate isomers are widely distributed in animal tissues as connective tissue or cell membrane components (Iozzo, 1985; Hewitt & Martin, 1984). Chondroitin 4-sulphate, chondroitin 6-sulphate and dermatan sulphate are the most well-known and abundant components. Dermatan sulphate was found to have an intimate relation to collagen fibre formation (Scott & Orford, 1981), and keratan sulphate PG and chondroitin sulphate PG have been shown to bind to specific sites on the collagen fibril (Scott & Haigh, 1985*a*, *b*; 1986), but the function and significance of each chondroitin sulphate isomer remains unclear.

A marked production of connective tissue is a well recognized phenomenon in a variety of invasive tumours. Connective tissues within cancer cell nests are induced from normal host tissues under the influence of tumour-derived factors, such as tumour growth factor, tumour angiogenic factor, and stimulating factor of glycosaminoglycan synthesis or collagen synthesis (Folkman & Haudenschild, 1980; Bano *et al.*, 1983; Iozzo, 1985; Ignotz & Massague, 1986), though some connective tissue proliferation is recognized as result of non-specific repair processes. Although the molecular mechanisms by which the induction of connective tissue is regulated are virtually unknown, changes of extracellular matrix components might be able to influence and facilitate the proliferation, infiltration and metastatic properties of the tumour cells. Chondroitin sulphate and hyaluronic acid in various tumour tissues are in much higher concentration than in non-neoplastic tissues (Takeuchi *et al.*, 1976; Dietrich *et al.*, 1977; Iozzo *et al.*, 1982*a*, *b*; Sobue *et al.*, 1987), and we proposed a potential role for chondroitin sulphate as a growth regulator in neoplasia (Takeuchi, 1965). Iozzo (1984) concluded that connective tissue rich in sulphated PG is a major determinant of the growth and infiltrative capacity of malignant cells.

Recently, the monoclonal antibodies 9A-2 and 3B-3 were produced against a stub of ΔDi-4S and ΔDi-6S unit binding to a core protein via a linkage tetrasaccharide obtained from chondroitin sulphate PG by chondroitinase ABC-treatment, respectively (Couchman *et al.*, 1984*a*; Caterson *et al.*, 1985). Using these antibodies, these authors demonstrated immunohistochemically a specific and distinctive distribution of chondroitin 4- or 6-sulphate PG in different connective tissue types; *viz.* chondroitin 6-sulphate distributions as revealed by the antibody 3B-3 after chondroitinase ABC pretreatment of tissue sections were observed not only in cartilage but also in the intima and media of aorta as well as basement membrane components, whereas chondroitin 4-sulphate as revealed by the antibody 9A-2 was found to be widely distributed in loose connective tissues of the normal rat. Recently, we obtained a monoclonal antibody against intact PG from the capsule of a human ovarian fibroma by a standard hybridoma technique in our laboratory. This antibody, designated 6B6, reacts specifically with the core molecules of dermatan sulphate PG (antigen). By staining with these antibodies, the localization of chondroitin sulphate and dermatan sulphate PG in tissue sections was achieved. In the present study, in order to clarify the major alterations of sulphated PG in tumour tissues, the identification of chondroitin sulphate PG and dermatan sulphate PG was studied by immunohistochemical techniques, using the antibodies (9A-2, 3B-3 and 6B6), on 70 surgically excised specimens. It was found that the distribution of PGs was different in the interstitial fibrous element of tumour tissues and normal tissues.

### Materials and methods

### *Antibodies*

Monoclonal antibodies used in the present study were: 9A-2 (against a stub of ΔDi-4S unit binding to core protein obtained from chondroitin sulphate PG by chondroitinase ABC) and 3B-3 (against a stub of ΔDi-6S unit binding to core protein obtained from chondroitin sulphate PG by chondroitinase ABC) (Couchman *et al.*, 1984*a*, *b*) and the gifts of Seikagaku Kogyo Company Ltd., Tokyo. Antibodies against dermatan sulphate PG, which was purified from the capsular tissue of a human ovarian fibroma (Sobue *et al.*, 1987), were produced by the conventional hybridoma

Correspondence: T. Fukatsu.

Received 5 May 1987; and in revised form, 17 July 1987.

Abbreviations: ΔDi-6S, 2-acetamide-2-deoxyl-3-O-(β-D-gluco-4-enepyranosyluronic acid)-6-O-sulfo-D-galactose; ΔDi-4S, 2-acetamide-2-deoxyl-3-O-(β-D-gluco-4-enepyranosyluronic acid)-4-O-sulfo-D-galactose.

technique in our laboratory. One of the monoclonal antibodies, IgG1, designated 6B6, was found to react specifically with the intact molecule of the antigen (dermatan sulphate PG) and the chondroitinase AC-treated core molecule on Western blotted nitrocellulose membrane.

Chondroitinase ABC and chondroitinase AC were purchased from Seikagaku Kogyo Co., Ltd., Tokyo.

*Tumour tissues*

The tumour tissues examined, which originated in our hospital, were: 33 carcinomas (6 adenocarcinomas of the stomach, 5 adenocarcinomas of the colon, 5 ductal carcinomas of the breast, 7 adenocarcinomas of the ovary, 4 endometrial carcinomas, 2 squamous cell carcinomas of the uterine cervix and 4 thyroid carcinomas), 1 malignant melanoma, 1 malignant poroma, 1 seminoma, 5 adenoid cystic carcinomas of the salivary gland, 5 fibroadenomas of the breast, and 24 non-epithelial tumours (5 leiomyomas, 8 neurogenic tumours, 2 chondrogenic tumours, 2 osteosarcomas, 3 malignant fibrous histiocytomas, 1 angioma and 1 liposarcoma and 2 hamartomas of the lung). Human normal tissues were also studied from 4 autopsy cases. Immediately after excision, tissue slices ~5 mm thick were cut from the excised tissues and fixed in glacial acetic acid 1% (v/v) in 95% ethanol (Sainte-Marie, 1962) at 4°C for 12–24 h. The specimens were dehydrated in an ethanol series of ascending concentrations and embedded in paraffin wax. Sections were 4 $\mu$m.

*Immunohistochemical detection of chondroitin sulphate PG and dermatan sulphate PG*

The staining methods for 9A-2 and 3B-3 were as follows: Deparaffinized sections were soaked in methanol containing 0.3% (v/v) $H_2O_2$, and then washed with PBS, followed by treatment with chondroitinase ABC. Digestion was done with 0.2 U ml$^{-1}$ of the enzyme in 20 mM Tris-HCl buffer pH 8.0 containing 20 mM acetic acid and protease inhibitors (10 mM EDTA, 10 mM phenylmethylsulfonylfluoride and 0.035 mM pepstatin, the same cocktail as described by Oike *et al.*, 1980). The reactions were performed at 37°C for 1 h. Enzyme-treated sections were allowed to react with normal goat serum (1:100 dilution), followed by reaction with the diluted mouse ascites (1:1000–2000) containing monoclonal antibody. After 1 h reaction of monoclonal antibody at room temperature, excess antibody was removed by washing with PBS. The bound antibodies were labelled with biotinylated anti-mouse immunoglobulin and peroxidase conjugated streptavidin (StrAviGen B-SA immunostaining kits, BioGENEX Lab. Dublin, Ca., USA). After washing again with PBS, tissue sections were soaked in 0.05 M sodium acetate-acetic acid buffer pH 5.0 containing 0.02% (w/v) 3-amino 9-ethylcarbazol and 0.014% (w/v) $H_2O_2$, and allowed to react for 5–10 min. Sections were counterstained with haematoxylin and embedded in gelatin solution. As controls, chondroitinase ABC-untreated tissue sections were employed. The staining method for 6B6 was the same as that for 9A-2 and 3B-3. For 6B6-staining, chondroitinase ABC treatment was not essential, but a small increase in intensity of positive reaction was observed in the treated sections. In the present study, the tissue sections were generally pretreated with chondroitinase ABC.

### Results

The localization of the substance revealed with each monoclonal antibody (9A-2, 3B-3 or 6B6) was compared among 70 tumour tissues and some normal tissues. The interstitial elements of all normal tissues were positive with 6B6 directed against the intact small dermatan sulphate PG: Dermis, submucosal layer of digestive canals, perichondral layer, perivascular connective tissue, adventitia of aorta, vascular endothelium, pleura, fibrous capsule of kidney and liver, endo- and peri-neurium of nerve sheath. Aortic wall and cartilage were not reactive with 6B6. The results suggest that antibody 6B6 reacts specifically with small dermatan sulphate PG-II, which was designated by Rosenberg *et al.* (1985). The epitope of the antibody 9A-2 is a stub of ΔDi-4S unit remaining on a core molecule of chondroitin sulphate PG and dermatan sulphate PG after chondroitinase ABC-treatment. Therefore, the reactive sites for the 6B6 were almost coincident with those for 9A-2 in normal interstitial tissues, except for cartilage and aorta. The epitope of antibody 3B-3 is a stub of ΔDi-6S unit remaining on a core molecule of chondroitin sulphate PG after chondroitinase ABC digestion. Antibody 3B-3 reacted with blood vessels, cartilage and basement membrane of some ductal cells. The sites reactive with 3B-3 were not coincident with those reactive either with 6B6 or 9A-2 in normal or neoplastic tissues.

*Epithelial tumours*

In most carcinoma tissues examined in the present study, the interstitial fibrous element, the so-called 'specific stroma' in cancer cell nests was observed to be intensely positive with antibody 9A-2, but it was negative or only weakly stained with 3B-3 and 6B6, as shown in Figures 1 & 2. The pre-existing connective tissue involved in the tumour growth and the fibrous tissue surrounding the cancer cell nests or capsules were positive with both 9A-2 and 6B6. These results indicated that the interstitial fibrous tissue of carcinoma is composed mainly of chondroitin 4-sulphate, whereas the supporting connective tissues and capsules contained PG consisting mainly of dermatan sulphate. With 3B-3, the intensely positive reaction was observed in the vessel walls, perivascular fibrous tissues and muscle tissues when they were invaded by carcinoma cells. Figure 3 shows that the blood vessels and perivascular connective tissue within carcinoma (cervical cancer) tissue was strongly positive with 3B-3. As shown in Figure 4, the connective tissue in association with gastric carcinoma cells in submucosa was positive with 3B-3, and the *muscularis mucosae* in the vicinity of the cancer cell nests was thickened and positive with 3B-3. These results may indicate that chondroitin 6-sulphate PG is synthesized by young connective tissues and muscle tissues when their proliferation is stimulated by adjacent tumour growth.

In the cases of adenoid cystic carcinoma, a unique staining pattern was observed (Figure 5a, b). The mucoid materials in the pseudocystic spaces were intensely stained with 3B-3 but not with 9A-2. On the contrary, the outer stromal areas were intensely reactive with 9A-2, but not 3B-3. Supporting connective tissue and capsule were positive with 6B6. The inner surface of pseudocyst and the intercellular material of the tumour cells were positive with the 3B-3. The interface between the tumour cell nests and the outer stromal areas were occasionally reactive for the 3B-3. As the pseudocystic spaces were replaced by hyalinized fibrous tissue, reactivity with 9A-2 could be observed while that with 3B-3 diminished.

In the cases of malignant poroma, malignant melanoma and seminoma, the thin fibrous tissues observed as the interstitial component in the tumour tissues were clearly positive with 9A-2, but not with 6B6 or 3B-3.

The myxomatous areas of intracanalicular fibroadenoma of the breast were stained only with 3B-3, while pericanalicular fibrous connective tissue was reactive with 9A-2 and 6B6. The basement membrane of ductal cells was occasionally positive with 3B-3 in cases of fibroadenoma and breast cancer (ductal carcinoma).

*Non-epithelial tumours*

In contrast to the epithelial tumours, positive reactions with 3B-3 were observed in the intercellular matrices in almost all

1

5a 5b

2

6

3

7

4

8

**Figure 1** Immunostaining with antibody 9A-2 in endometrial carcinoma. The interstitial fibrous element is intensely positive. (Counterstained with haematoxylin, ×75). **Figure 2** Immunostaining with antibody 6B6 in the same section as **Figure 1**. The interstitial fibrous element is very weakly positive. (Counterstained with haematoxylin, ×75). **Figure 3** Immunostaining with antibody 3B-3 in squamous cell carcinoma of the uterine cervix. Intensely positive reaction is seen in the stromal element of vessel wall and the perivascular fibrous tissues within cancer cell nest. (Counterstained with haematoxylin, ×75). **Figure 4** Immunostaining with antibody 3B-3 in gastric cancer. Proliferating connective tissue in association with the invasive growth of carcinoma cells is positive. The positive reaction is also seen in the thickened *muscularis mucosae* in the vicinity of the cancer cell nests and in the stromal element of blood vessel. (Counterstained with haematoxylin, ×15). **Figure 5** Immunostaining with antibodies 3B-3 (*a*) and 9A-2 (*b*) in adenoid cystic carcinoma. The 3B-3 positive reaction is seen in the mucoid substance in the pseudocystic spaces, and occasionally in the interface between outer stromal area and cancer cell nests (*a*). The 9A-2 positive reaction is seen in the outer stromal areas (*b*). (Counterstained with haematoxylin, ×30). **Figure 6** Immunostaining with antibody 3B-3 in malignant fibrous histiocytoma of the bone (femur). The stromal element is observed to be positive. (Counterstained with haematoxylin, ×75). **Figure 7** Immunostaining with antibody 3B-3 in osteosarcoma. The stromal element of chondroid areas is strongly positive. (Counterstained with haematoxylin, ×75). **Figure 8** Immunostaining with antibody 6B6 in the same section as **Figure 7**. The hyalinous collagen and osteoid are positive. (Counterstained with haematoxylin, ×75).

cases (fibrogenic, myogenic, chondrogenic and osteogenic tumours). Figure 6 shows that 3B-3 positive reaction in an area showing a storiform pattern of malignant fibrous histiocytoma. In general, the portions where collagenization became marked were positive with 9A-2, and when the fibrous components became hyalinized, the reaction with both 9A-2 and 6B6 was intense.

Chondroid tissues, both benign and malignant, were strongly positive with 3B-3. In a case of osteosarcoma, a typical cartilage formation was observed in association with deposition of hyalinized collagen and osteoid. Figures 7 & 8 show that the neoplastic cartilage was strongly positive with 3B-3 in contrast to the intensely positive reaction with 6B-6 in hyalinized collagen and osteoid. In a hamartoma occurring in the lung, chondromatous tissue and loose connective tissue consisting of immature mesenchymal cells were positive with 3B-3.

### Discussion

Antibodies 9A-2 and 3B-3 react with the 4- and 6-sulphated disaccharide units, respectively, which are generated from the chondroitin sulphate chains by digestion with chondroitinase ABC. The antibodies do not recognize the entire constituency of the chondroitin sulphate-repeating units. Chondroitinase ABC, which is an endo-eliminase, can degrade the chondroitin sulphate chain so as to leave only one disaccharide unit attached to the linkage tetrasaccharide (Oike *et al.*, 1980). The antibodies probably react to the disaccharide units bound intimately to the linkage tetrasaccharides. Couchman *et al.* (1984*a*, *b*) showed that in addition to chondroitin 4-sulphate, dermatan sulphate was also stained with 9A-2 in rat cartilage, dermis and loose connective tissue after chondroitinase ABC digestion.

It has been reported that the amount of glycosaminoglycan in tumour tissues is much larger than in non-neoplastic tissues (Kojima *et al.*, 1975; Dietrich *et al.*, 1977; Takeuchi *et al.*, 1976; Sobue *et al.*, 1980*b*; Sobue *et al.*, 1987; Iozzo *et al.*, 1982*a*, *b*). Recently, we demonstrated that the glycosaminoglycan-synthetic activity of tumour tissues was markedly higher than that of non-neoplastic tissues in human gastric mucosa and adipose tissues (Sobue *et al.*, 1980*a*, *b*). We also demonstrated that leiomyosarcoma contains a large amount of large PG with chondroitin sulphate side chains and small PG having dermatan sulphate-chondroitin sulphate side chains (Sobue *et al.*, 1987). The core molecule of the small PG from leiomyosarcoma was quite similar in molecular weight to those from benign tumours (leiomyoma, ovarian fibroma and Schwannoma). The small PGs from the benign tumours are considered to be of the same PG family as those from non-neoplastic tissues such as skin (Damle *et al.*, 1982), uterine cervix (Uldbjerg *et al.*, 1983), bone (Franzen & Heinegard, 1984), tendon (Vogel & Heinegard, 1985), cartilage (Rosenberg *et al.*, 1986) and other tissues. Antibody 6B6 was also found to react with the core molecules obtained from the small PG of benign non-epithelial tumours as described in a previous paper (Sobue *et al.*, 1987). The present results showed that the interstitial fibrous element of carcinoma was positive with 9A-2, but negative or very weakly positive with 6B6. This presumably indicates that the interstitial fibrous element of carcinoma tissue is composed mainly of chondroitin 4-sulphate PG with only a little, if any, dermatan sulphate PG. It is conceivable that the interstitial elements of carcinoma tissues contain PG having the core molecule other than the small PG revealed with 6B6 or a core molecule modified at the epitope site of reactivity with 6B6, though the PG in the surrounding connective tissue is of the same small PG family as described above.

It has been observed that cultured myogenic cells or embryonic myogenic tissues produce high buoyant density PGs carrying predominantly chondroitin 6-sulphate side chains (Carrino & Caplan, 1982; 1984). Recently, Hollman *et al.* (1986) found that proliferating arterial smooth muscle cells produce PG, consisting mainly of chondroitin 6-sulphate, at a higher rate than non-dividing cells. Wight *et al.* (1985) demonstrated that smooth muscle cells increase sulphated PG synthesis ~3–4 fold when stimulated to proliferate. In the present study, a markedly positive reaction for antibody 3B-3 was observed in the stromal element of blood vessels proliferating within or near the carcinoma cell nests. The connective tissues growing from the muscle layer, *muscularis mucosae* and vessel walls in association with the infiltrative growth of carcinoma cells were also intensely positive with 3B-3. These results suggest that the PG carrying chondroitin 6-sulphate is synthesized by the myogenic cells which are proliferating in association with tumour growth. It is conceivable that the growing tumour cells stimulate the proliferation of connective tissue cells from muscle tissue whose chondroitin 6-sulphate synthetic activity is markedly enhanced. Since it has been reported that transforming growth factor or connective tissue activating peptide (platelet derived growth factor) stimulate synthesis of extra-cellular matrix components (Castor *et al.*, 1983; Ignotz & Massague, 1986), it is conceivable that these factors act on chondroitin 6-sulphate-synthesis and on proliferating connective tissue in association with tumour growth.

The present result also indicated that adenoid cystic carcinoma cells produce PG consisting mainly of chondroitin 6-sulphate which accumulated in the pseudocystic spaces, being replaced by chondroitin 4-sulphate if hyalinization progressed. Toida *et al.* (1985) reported that the pseudocysts of adenoid cystic carcinoma represent a peculiar structure consisting of basement membrane components. Couchman *et al.* (1984*a*), using antibody 3B-3, demonstrated a striking specificity of staining in basement membrane zones. They had confirmed lack of cross-reactivity of the 3B-3 antibody with other basement membrane components (laminin, type IV collagen and heparan sulphate PG) by antigen absorption of ELISA assays. On the other hand, it is well known that myoepithelial cells contribute to the formation of the unique structure of adenoid cystic carcinoma (Hübner *et al.*, 1971; Hoshino & Yamamoto, 1970). Therefore, the present results seemed to show that the appearance of chondroitin 6-sulphate in adenoid cystic carcinoma had an intimate relationship with the proliferation of myoepithelial cells which are able to form a large amount of the basement membrane components.

In the present study, 3B-3-positive reactivity was also observed in matrix-rich connective tissues, such as the myxomatous areas of intracanalicular fibroadenoma of the breast, the loose connective tissue in lung hamartoma and the myxoid areas of non-epithelial tumours, whereas 9A-2-positivity was observed in the fibrous connective tissue and when collagenization progressed the reactivity with both 9A-2 and 6B6 was observed to be more intense. It was conceivable that the presence of chondroitin 6-sulphate has an antifibrotic action, whereas chondroitin 4-sulphate has a close connection with collagenization of fibrous connective tissue.

We acknowledge the financial support of a grant from the Ministry of Education, Science and Culture, Japan. The technical assistance of Chikage Yasui and Mari Niwa is gratefully acknowledged.

### References

BANO, M., ZWIEBEL, J.A., SALOMON, D.S. & KIDWELL, W.R. (1983). Detection and partial characterization of collagen synthesis stimulating activities in rat mammary adenocarcinomas. *J. Biol. Chem.*, **258**, 2729.

CARRINO, D.A. & CAPLAN, A.I. (1982). Isolation and preliminary characterization of proteoglycans synthesized by skeletal muscle. *J. Biol. Chem.*, **257**, 14145.

CARRINO, D.A. & CAPLAN, A.I. (1984). Isolation and partial characterization of high-buoyant-density proteoglycans synthesized in ovo by embryonic chick skeletal muscle and heart. *J. Biol. Chem.*, **259**, 12419.

CASTOR, C.W., MILLER, J.W. & WALZ, D.A. (1983). Structural and biological characteristics of connective tissue activating peptide (CTAP), a major human platelet-derived growth factors. *Proc. Natl Acad. Sci. USA*, **80**, 765.

CATERSON, B., CHRISTNER, J.E., BAKER, J.R. & COUCHMAN, J.R. (1985). Production and characterization of monoclonal antibodies directed against connective tissue proteoglycans. *Fed. Proc.*, **44**, 386.

COUCHMAN, J.R., CATERSON, B., CHRISTNER, J.E. & BAKER, J.R. (1984*a*). Mapping monoclonal antibody detection of glycosaminoglycans in connective tissues. *Nature*, **307**, 650.

COUCHMAN, J.R., CATERSON, B., CHRISTNER, J.E. & BAKER, J.R. (1984*b*). Chondroitin sulphate proteoglycans in extracellular matrices – Sulphation specificity in relation to cellular differentiation. In *Matrices and Cell Differentiation*, Kemp, R.B. & Hinchliffe, J.R. (eds) p. 31. Alan R. Liss Inc., New York.

DAMLE, S.P., COSTER, L. & GREGORY, J.D. (1982). Proteodermatan sulfate isolated from pig skin. *J. Biol. Chem.*, **257**, 5523.

DIETRICH, C.P., SAMPAIO, L.O., TOLEDO, O.M.S. & CASSARO, C.M.F. (1977). Recognition and adhesiveness: A possible biological role for the sulfated mucopolysaccharides. *Biochem. Biophys. Res. Comm.*, **75**, 329.

FOLKMAN, J. & HAUDENSCHILD, C. (1980). Angiogenesis *in vitro*. *Nature*, **288**, 551.

FRANZEN, A. & HEINEGARD, D. (1984). Characterization of proteoglycan from the calcified matrix of bovine bone. *Biochem. J.*, **224**, 59.

HEWITT, A.T. & MARTIN, G.R. (1984). Attachment protein and their role in extracellular matrices. In *The Biology of Glycoproteins*, Ivatt, R.J. (ed) p. 65. Plenum Press: New York.

HOLLMANN, H., THIEL, J., SCHMIDT, A. & BUDDECKE, E. (1986). Increased activity of chondroitin sulfate-synthesizing enzymes during proliferation of arterial smooth muscle cells. *Exptl. Cell Res.*, **167**, 484.

HOSHINO, M. & YAMAMOTO, I. (1970). Ultrastructure of adenoid cystic carcinoma. *Cancer*, **25**, 180.

HÜBNER, G., KLEIN, H.J., KLEINSASSER, O. & SCHIEFER, H.G. (1971). Role of myoepithelial cells in the development of salivary gland tumors. *Cancer*, **27**, 1255.

IGNOTZ, R.A. & MASSAGUE, J. (1986). Transforming growth factor-$\beta$ stimulates the expression of fibronectin and collagen and their incorporation into the extracellular matrix. *J. Biol. Chem.*, **261**, 4337.

IOZZO, R.V. & WIGHT, T.N. (1982*a*). Isolation and characterization of proteoglycans synthesized by human colon and colon carcinoma. *J. Biol. Chem.*, **25**, 11135.

IOZZO, R.V., BOLENDER, R.P. & WIGHT, T.N. (1982*b*). Proteoglycan changes in the intercellular matrix of human colon carcinoma. An integrated biochemical and stereologic analysis. *Lab. Invest.*, **47**, 124.

IOZZO, R.V. (1984). Proteoglycans and neoplastic-mesenchymal cell interactions. *Hum. Pathol.*, **15**, 2.

IOZZO, R.V. (1985). Biology of disease. Proteoglycans: Structure and function, and role in neoplasia. *Lab. Invest.*, **53**, 373.

KOJIMA, J., NAKAMURA, N., KANATANI, M. & OHMORI, K. (1975). The glycosaminoglycan in human hepatic cancer. *Cancer Res.*, **35**, 542.

OIKE, Y., KIMATA, K., SHINOMURA, T., NAKAZAWA, K. & SUZUKI, S. (1980). Structural analysis of chick-embryo cartilage proteoglycan by selective degradation with chondroitin lyases (chondroitinase) and endo-$\beta$-D-galactosidase (keratanase). *Biochem. J.*, **191**, 193.

ROSENBERG, L.C., CHOI, H.U., TANG, L.-H. & 4 others (1986). Isolation of dermatan sulfate proteoglycans from mature bovine articular cartilages. *J. Biol. Chem.*, **260**, 6304.

SAINTE-MARIE, G. (1962). A paraffin embedding technique for studies employing immunofluorescence. *J. Histochem. Cytochem.*, **10**, 250.

SCOTT, J.E. & ORFORD, C.R. (1981). Dermatan sulphate-rich proteoglycan associates with rat tail-tendon collagen at the D band in the gap region. *Biochem. J.*, **197**, 213.

SCOTT, J.E. & HAIGH, M. (1985*a*). Proteoglycan-type I collagen fibril interactions in bone and non-calcifying connective tissues. *Biosci. Rep.*, **5**, 71.

SCOTT, J.E. & HAIGH, M. (1985*b*). 'Small'-proteoglycan: Collagen interactions: Keratan sulphate proteoglycan associates with rabbit corneal collagen fibrils at the 'a' and 'c' bands. *Biosci. Rep.*, **5**, 765.

SCOTT, J.E. & HAIGH, M. (1986). Prtoteoglycan-collagen interactions in intervertebral disc. A chondroitin sulphate proteoglycan associates with collagen fibrils in rabbit annulus fibrosus at the d–e bands. *Biosci. Rep.*, **6**, 879.

SOBUE, M., MIURA, K., KATAOKA, K., TSUJI, K. & TAKEUCHI, J. (1980*a*). Glycosaminoglycan-synthetic activity of neoplastic and non-neoplastic adipose tissues. *Br. J. Cancer*, **42**, 477.

SOBUE, M., TAKEUCHI, J., MIURA, K., KAWASE, K., MIZUNO, F. & SATO, E. (1980*b*). Glycosaminoglycan content and synthesis in gastric carcinoma. *Br. J. Cancer*, **42**, 78.

SOBUE, M., TAKEUCHI, J., YOSHIDA, K. & 4 others (1987). Isolation and characterization of proteoglycans from human non-epithelial tumors. *Cancer Res.*, **47**, 160.

TAKEUCHI, J. (1965). Growth-promoting effect of chondroitin sulphate on solid Ehrlich ascites tumour. *Nature*, **207**, 537.

TAKEUCHI, J., SOBUE, M., SATO, E., SHAMOTO, M., MIURA, K. & NAKAGAKI, S. (1976). Variation in glycosaminoglycan components of breast tumors. *Cancer Res.*, **36**, 2133.

TOIDA, M., TAKEUCHI, J., SOBUE, M. & 4 others (1985). Histochemical studies on pseudocysts in adenoid cystic carcinoma of the human salivary gland. *Histochem. J.*, **17**, 913.

ULDJERG, N., MALMSTROM, A., EKMAN, G., ULMSTEN, U. & WINGERUP, L. (1983). Isolation and characterization of dermatan sulphate proteoglycan from human uterine cervix. *Biochem. J.*, **209**, 497.

VOGEL, K.G. & HEINEGARD, D. (1985). Characterization of proteoglycans from adult bovine tendon. *J. Biol. Chem.*, **260**, 9298.

WIGHT, T.N., KINSELLA, M.G. & POTTER-PERIGO, S. (1985). Proteoglycans synthesized and secreted by cultured vascular cells. In *Extracellular Matrix: Structure and Function*, Reddi, A.H. (ed) p. 321. Alan R. Liss: New York.